# Mediterranean Spotted Fever: A Rare Non-Endemic Disease in the USA

**DOI:** 10.7759/cureus.974

**Published:** 2017-01-12

**Authors:** Joshua Brad Oaks, Glenmore Lasam, Gina LaCapra

**Affiliations:** 1 Department of Medicine, Overlook Medical Center

**Keywords:** mediterranean spotted fever, boutonneuse fever, brown dog tick, rickettsia conorii, tache noire, doxycycline

## Abstract

We report a case of a 43-year-old Israeli male who presented with an intermittent fever associated with a gradual appearance of diffusely scattered erythematous non-pruritic maculopapular lesions, generalized body malaise, muscle aches, and distal extremity weakness. He works in the Israeli military and has been exposed to dogs that are used to search for people in tunnels and claimed that he had removed ticks from the dogs. In the hospital, he presented with fever, a diffuse maculopapular rash, and an isolated round black eschar. He was started on doxycycline based on suspected Mediterranean spotted fever (MSF) in which he improved significantly with resolution of his clinical complaints. His immunoglobulin G (IgG) MSF antibody came back positive.

## Introduction

Mediterranean spotted fever (MSF) is a rare tick-borne disease in the United States and has been imported from endemic areas. A comprehensive health history including travel and exposure elucidated the dilemma of the myriads of differentials in patients presenting with a fever and a rash.

## Case presentation

A 43-year-old Israeli male with diabetes mellitus presented with fever and rash for almost 10 days which occurred while traveling across the country as a tourist. He had started off first with an intermittent fever then myalgia. He continued to have fever and chills associated with night sweats, with the spread of a non-pruritic rash from his hands, trunk, and feet, and worsening myalgia. He had difficulty in ambulating because of prostration. He had recently traveled and his vaccines were up to date. He did not state that he had any history of mosquito bites and his symptoms started even before he left his country. He consulted two physicians without a definite diagnosis. He eventually developed muscle aches and distal weakness in all his four extremities, which then prompted consult to the hospital. Upon further inquiry of travel or tick bite exposure, the patient revealed that he works in the Israeli military with dogs that are used to search an underground passage for people before collapsing the tunnels. He claimed that he pulled ticks off the dogs.

**Figure 1:**
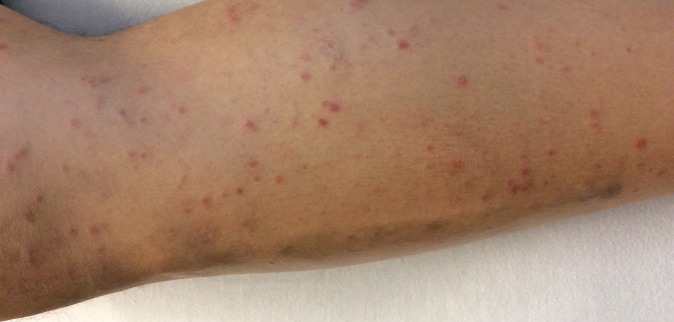
An erythematous non-pruritic maculopapular rash scattered diffusely on the patient’s leg.

**Figure 2:**
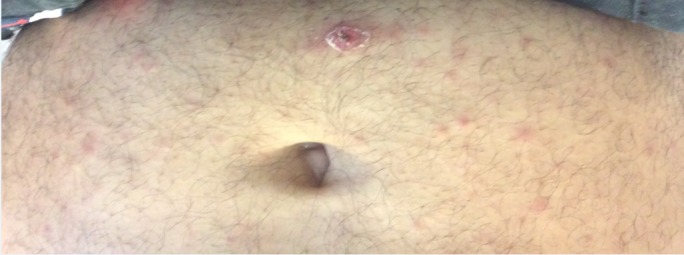
A 1 cm black eschar with a surrounding erythema, which is called ‘tache noire,’ in the umbilical area in a patient with Mediterranean spotted fever.

A physical examination revealed a fever of 101.5°F, a diffuse maculopapular rash on his body including his palms and soles but sparing the head and neck (Figure 1), and a 1 cm round black eschar with surrounding erythema noted below the umbilicus (Figure 2). He had diffuse tenderness on palpation of his four extremities. Hemogram, comprehensive metabolic panel, chest radiograph, and cerebrospinal fluid analysis were unremarkable. Serological tests to detect Anaplasma phagocytophilum, Ehrlichia chaffeensis, Babesia microti, rapid plasma reagin, lyme disease, West Nile virus, dengue virus, Epstein-Barr virus, parvovirus, and human immunovirus antibodies were all negative.

Based on the patient’s history, dermatological findings, and epidemiologic disease prevalence in Israel, we suspected MSF and started him on doxycycline. He improved significantly with lysis of fever, resolution of body malaise and extremity weakness, and improvement of his skin lesions. The Rickettsia conorii IgG came back positive. Informed consent was obtained from the patient for this study.

## Discussion

MSF or boutonneuse fever is caused by Rickettsia conorii, an obligate intracellular bacterium. The disease ensues when humans are bitten by an infected vector, the brown dog tick Rhipicephalus sanguineus [1]. MSF is endemic in the Mediterranean, regions around the Black Sea, and the Sub-Saharan African countries; however, sporadic cases have been reported in non-endemic areas either in travelers or in patients with contact to infected dogs from endemic places [2]. MSF is uncommon in the United States with less than 50 imported cases that have been clinically and serologically identified by the Centers for Disease Control and Prevention (CDC) [1]. This might be attributed to the non-reportability of the disease as well as the physician’s inadequate recognition of the clinical features and the disease epidemiology.

The disease presents with an abrupt onset of headache, fever, [2] and a maculopapular rash which may be petechial in 10% of cases [3]. The maculopapular rash is present in 97–99% of cases, and an eschar or black necrotic scabbed lesion, which is called a ‘tache noire,’ is seen at the site of the inoculating tick bite in 70% of cases [3-4].

Differential diagnosis of the spotted fever group (SFG) rickettsiae aside from MSF includes Rocky Mountain spotted fever (Rickettsia rickettsii ), Queensland tick typhus (Rickettsia australis), Flinders Island spotted fever (Rickettsia honei), African tick bite fever (Rickettsia africae), Siberian tick typhus (Rickettsia sibirica), Japanese spotted fever (Rickettsia japonica), Flea rickettsiosis (Rickettsia felis), Rickettsia slovaca infection (Rickettsia slovaca), Rickettsia parkeri infection (Rickettsia parkeri), Rickettsia amblyommii infection (Rickettsia amblyommii), and the recent Rickettsia 364D infection (Rickettsia 364D) among others [2].

MSF is diagnosed based on clinical manifestations, epidemiologic data, and laboratory evidence of recent exposure to rickettsial organisms in which both culture techniques and serologic tests are used to confirm the diagnosis; however, indirect immunofluorescence (IIF) is the most commonly used confirmatory test currently [5]. A titer of 1:64 or greater is diagnostic [6].

Antimicrobial therapy should not be held pending diagnostic confirmation in a patient with a suspected disease compatible with the history and physical examination findings. The preferred drug treatment is doxycycline for seven days to fourteen days while erythromycin in pregnant women is not as effective as the tetracyclines [5].

The limited data on MSF deaths accentuated a solitary literature on the mortality rate of 21% (29 out of 140 patients from 13 hospitals) on documented MSF in hospitalized Portuguese patients with 29% (20 patients) case fatality ratio for the Israeli spotted fever strain, known as R. conorii israelensis, compared to 13% (nine patients) for those infected with the Malish strain [7].

## Conclusions

Many patients present with fever and rash that may suggest a wide variety of possible etiologies. However, the importance of obtaining a thorough travel and occupational history as in this case would assist in clinching the diagnosis of rare tick-borne disease that is non-endemic in this country.
